# Predictors of resignation and sick leave after cancer diagnosis among Japanese breast cancer survivors: a cross-sectional study

**DOI:** 10.1186/s12889-021-10168-2

**Published:** 2021-01-14

**Authors:** Kiyomi Mitsui, Motoki Endo, Yuya Imai, Yuito Ueda, Hiroko Ogawa, Go Muto, Yan Yan, Gautam A. Deshpande, Yasuhisa Terao, Satoru Takeda, Takeshi Tanigawa, Katsuji Nishimura, Kazuhiko Hayashi, Mitsue Saito, Akatsuki Kokaze

**Affiliations:** 1 Department of Hygiene, Public Health, and Preventive Medicine, Showa University, Tokyo, Japan; 2 Department of Public Health, Juntendo University Faculty of Medicine, Tokyo, Japan; 3 Department of Hygiene, Kitasato University School of Medicine, Sagamihara, Kanagawa Japan; 4 Department of Palliative Medicine, Juntendo University Graduate School of Medicine, Tokyo, Japan; 5 Department of General Medicine Juntendo University, Tokyo, Japan; 6 Department of Obstetrics and Gynecology, Juntendo University Faculty of Medicine, Tokyo, Japan; 7 Department of Psychiatry, Tokyo Women’s Medical University School of Medicine, Tokyo, Japan; 8 Department of Chemotherapy and Palliative Care, Tokyo Women’s Medical University Hospital, Tokyo, Japan; 9 Department of Breast Oncology, Juntendo University School of Medicine, Tokyo, Japan

**Keywords:** Breast cancer survivors, Resignation, Sick leave, Return to work

## Abstract

**Background:**

In Japan, 55.5% of breast cancer survivors (BCSs) are of working age, so various perspectives regarding return to work (RTW) after cancer diagnosis need to be considered. Therefore, this study aimed to clarify the risk factors for resignation and taking sick leave (SL) among BCSs in continued employment at the time of diagnosis.

**Methods:**

A web-based retrospective cross-sectional survey was conducted on BCSs using data from a 2018 Japanese national research project (Endo-Han) commissioned by the Ministry of Health, Labour and Welfare of Japan. The subjects were women aged 18–69 years who had been diagnosed with breast cancer for the first time at least 1 year previously. The risk factors for resignation and taking SL after breast cancer diagnosis, including age at diagnosis, education level, cancer stage, surgery, chemotherapy, radiotherapy, employment status, and occupational type, were then analyzed using a logistic regression model.

**Results:**

In total, 40 (14.9%) of 269 BCSs quit their jobs at least 1 year after being diagnosed with breast cancer. The results of the multivariable analysis indicated that lower education level (odds ratio [OR]: 3.802; 95% confidence interval [CI]: 1.233–11.729), taking SL (OR: 2.514; 95%CI: 1.202–5.261), and younger age at diagnosis (OR: 0.470; 95%CI: 0.221–0.998) were predictors of resignation. Of 229 patients who continued working, SL was taken by 72 (31.4%). In addition, undergoing surgery was found to be a predictor of taking SL (OR: 8.311; 95%CI: 1.007–68.621).

**Conclusions:**

In total, 40 (14.9%) of 269 BCSs quit their jobs at least 1 year after being diagnosed with breast cancer. The results of this study indicated that younger age, lower education level, and taking SL were predictors of resignation after breast cancer diagnosis.

**Supplementary Information:**

The online version contains supplementary material available at 10.1186/s12889-021-10168-2.

## Background

The number of breast cancer survivors (BCSs), along with their 5-year survival rates, continue to rise steadily in Japan because of early detection and advances in treatment [1]. As breast cancer survival rates have increased, issues surrounding the quality of life (QOL) of BCSs, including palliative care, mental health, and employment, have received more attention [2–5]. In 2015, approximately 55.5% of the 83,959 BCSs in Japan belonged to a working age group, typically defined as 20–64 years old [6]. As the number of working women has been increasing in Japan [7], it is expected that more working-age women will be diagnosed with breast cancer in the near future, following trends seen in Western countries [8–10]. In Japan, it seems there has been more interest in striking a balance between cancer treatment and work [11]. In 2016, the Japanese government amended the Cancer Control Act (this law sets out a duty for employers to strive to keep cancer survivors [CSs] working) and published guidelines outlining support for individuals undergoing therapy during working life to aid employers in providing better support to employees with cancer, similar to the Netherlands [11].

Maintaining employment after breast cancer diagnosis remains an important issue for not only BCSs and their families, but also employers and society [12]. Previous studies suggest that maintaining employment after breast cancer diagnosis is affected by three primary domains: personal factors (e.g., age, sex, education), clinical factors (e.g., cancer site, cancer stage), and work-related factors (e.g., company size, social support resources) [9, 12, 13]. Return to work (RTW) after cancer diagnosis is undoubtedly challenging for a variety of reasons, including physical symptoms (e.g., cancer-related fatigue, pain, hair loss, nausea) [14]; however, unemployment (not working) after breast cancer diagnosis has also been shown to reduce QOL [2–4], and previous studies have found that BCSs are more likely to be unemployed [15, 16]. As a contributing factor, breast cancer has been shown to be associated with long RTW times, as well as a lower cumulative RTW rate, compared with individuals with gastric or female genital cancer [17].

Moreover, predictors of work resignation (quitting work) among BCSs include contract or part-time work, with these types of workers demonstrating higher odds of resignation compared with regular and full-time workers [18]. However, the relationship between resignation and treatment modality or individual factors has not been fully clarified, and less attention has been paid to predictors of resignation and sick leave (SL) among BCSs in Japan. In Japan, BCSs who remain on SL often seem to experience financial difficulties because after using up their paid leave, they only receive more than 60% of their salary as a sickness allowance during SL [19].

Given this background, the objective of this study was to clarify the predictors of resignation and SL among BCSs in continued employment. Clarifying these predictors could be expected to aid health care providers in supporting CSs who continue to work, and to provide evidence that assists physicians, health care staff, and employers in establishing and improving work support systems for BCSs [20].

## Methods

### Study participants

A web-based retrospective cross-sectional survey was conducted on BCSs using data from a 2018 Japanese national research project (Endo-Han) commissioned by the Ministry of Health, Labour and Welfare (MHLW) of Japan. The project developed a questionnaire asking for information about the following factors: age at time of diagnosis; education level; cancer stage; treatment, including surgery, cancer chemotherapy, and radiotherapy; employment status and type (permanent vs. non-permanent work); occupational type (office worker vs. non-office worker); and history of SL use and resignation. Women aged 18–69 years who had been diagnosed with breast cancer for the first time at least 1 year previously were eligible for participation. On January 17–18, 2018, an online questionnaire (in Japanese) was sent via e-mail to 4968 BCSs (age range: 18–69 years) who had registered with the commercial cancer panel Macromill (www.macromill.com/global/index.html). The reward for answering was in the form of points according to the number of questions answered; these points could then be redeemed as cash or exchanged for items.

**Fig. 1:**
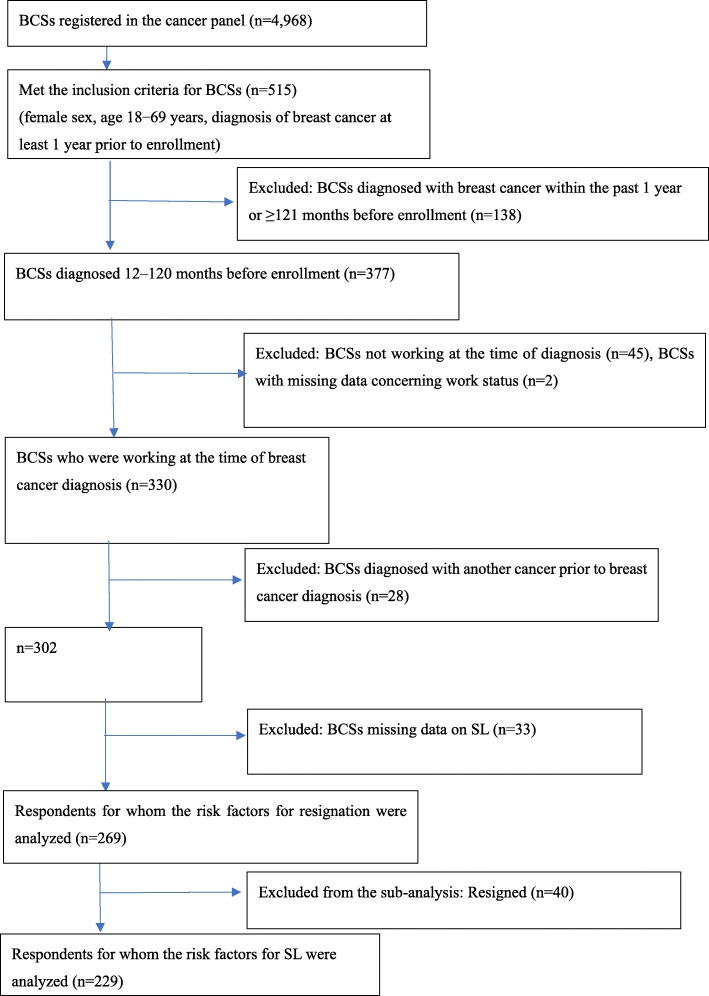
Flowchart of patient enrollment. BCS, breast cancer survivor; SL, sick leave

The inclusion criteria were female sex, age 18–69 years, and first breast cancer diagnosis occurring at least 1 year previously. Participants who had been diagnosed with breast cancer within the past 1 year or ≥ 121 months (1–10 years after the date of breast cancer diagnosis) (*n* = 138), were not working at the time of breast cancer diagnosis (*n* = 45), had missing data (*n* = 2), had a history of cancer other than breast cancer (*n* = 28), or provided unclear answers regarding SL (*n* = 33) were excluded (Fig. 1). Finally, 269 respondents were subjected to analysis. The response rate (10.4%; 515/4968 breast cancer patients) was relatively low. However, this response rate was almost similar to that observed in a previous study using the same research company [20]. Subgroup analyses of predictors of SL excluded 40 patients who resigned without taking SL. Patients who reported continuing to work after breast cancer diagnosis without taking any time off and those reporting RTW following diagnosis after taking time off using annual paid vacation allowance (annual leave) were classified into the “no SL” group, whereas patients who reported RTW after taking time off for recuperation using SL, unscheduled absences, or leaves of absence due to insufficient annual paid vacation time were classified into the “SL” group.

The following terms are generally defined as follows in Japan: RTW: returning to the workforce, resignation: leaving a job, SL: after workers use up their paid leave, this generally becomes known as SL; during SL, employees are paid > 60% of their usual remuneration by their health insurance in accordance with the Labor Standards Act of Japan [19].

### Statistical analysis

Risk factors for resignation were analyzed after classifying the patients into continued employment vs. resignation groups. The chi-squared test and multiple logistic regression analysis were performed with the following covariate independent variables: age at time of diagnosis (median: < 47 vs. ≥47 years), educational attainment (higher education level [i.e., university, graduate school] vs. lower education level [i.e., high school, vocational school, junior college]); cancer stage (early [0, I] vs. advanced [II–IV]); surgery (yes/no); cancer chemotherapy (yes/no); radiotherapy (yes/no); employment status (permanent vs. nonpermanent [i.e., contract, temporary, other]); occupation type (office work vs. non-office work); and SL (yes/no). The dependent variable was continued employment vs. resignation. Multiple logistic regression analysis including all variables was performed to analyze the risk factors for resignation.

In a sub-analysis of the continued employment group, risk factors for taking SL were explored after classifying the patients into no SL vs. SL groups. To analyze the risk factors for SL, the chi-squared test and multiple logistic regression analysis including all variables were performed using the above covariate independent variables, with SL (yes/no) as the dependent variable. Data were analyzed using SPSS Statistics for Windows ver. 25 (IBM Corp. Armonk, NY, USA), with significance for all tests set at *p* < 0.05. The need for informed consent was waived in line with ethical guidelines in medical and health research involving human subjects in Japan [21]. This study was approved by the Juntendo University Ethics Review Committee (Approval No. 2018042). The medical ethics committee of Juntendo University informed us that informed consent was not required because the previously existing data were anonymous and impossible to concatenate; therefore, no associated correspondence table is provided, in accordance with national guidelines [21].

## Results

Of the 269 BCSs analyzed, 40 (14.9%) resigned from their jobs after being diagnosed with cancer (Table 1). Median age at the time of cancer diagnosis was 46.0 years (range: 19–69; age < 47 years (*n* = 143 [53.2%]); age: ≥47 years (*n* = 126 [46.8%]). Mean duration from breast cancer diagnosis to the date of the survey was 55.9 months (approximately 4.5 years). In addition, 73 BCSs (27.1%) had a higher education level, and 163 (60.6%) had early-stage cancer. Regarding treatment methods, 250 (92.9%), 199 (74.0%), and 174 (64.7%) BCSs had experienced surgery, cancer chemotherapy, and radiotherapy, respectively. Regarding occupation type, 117 (43.5%) and 160 (59.5%) BCSs were permanent and desk workers, respectively, and 95 (35.3%) had taken SL.

**Table 1:** Basic characteristics of the analyzed respondents (*n* = 269)

Variable	Resigned (*n* = 40)	Not resigned (*n* = 229)	*p*-value
	n (%)	n (%)	
Age at time of diagnosis, y			
< 47	26 (65.0)	117 (51.1)	0.104
≥ 47	14 (35.0)	112 (48.9)	
Education level			
Higher (university, graduate school)	4 (10.0)	69 (30.1)	0.007**
Lower (high school, vocational school, junior college)	36 (90.0)	160 (69.9)	
Cancer stage			
Early (0, I)	18 (45.0)	145 (63.3)	0.029*
Advanced (II–IV)	22 (55.0)	84 (36.7)	
Surgery			
No	1 (2.5)	18 (7.9)	0.325
Yes	39 (97.5)	211 (92.1)	
Chemotherapy			
No	9 (22.5)	61 (26.6)	0.582
Yes	31 (77.5)	168 (73.4)	
Radiotherapy			
No	17 (42.5)	78 (34.1)	0.303
Yes	23 (57.5)	151 (65.9)	
Type of employment			
Permanent	17 (42.5)	100 (43.7)	0.891
Non-permanent	23 (57.5)	129 (56.3)	
Occupation type			
Office work	17 (42.5)	143 (62.4)	0.018*
Non-office work	23 (57.5)	86 (37.6)	
Sick leave			
No	17 (42.5)	157 (68.6)	0.001**
Yes	23 (57.5)	72 (31.4)	

* < 0.05, ** < 0.01

As shown in Table 2, multivariable logistic regression analysis regarding risk factors for resignation identified significant odds ratios (ORs) for the following three factors: lower education level (OR: 3.802; 95% confidence interval [CI]: 1.233–11.729; *p* = 0.020), taking SL (OR: 2.514; 95%CI: 1.202–5.261; *p* = 0.014), and age ≥ 47 years (OR: 0.470; 95%CI: 0.221–0.998).

**Table 2:** Univariable and multivariable logistic regression analysis regarding risk factors for resignation

		Univariable		Multivariable	
		OR (95% CI)	*p*-value	OR (95% CI)	*p*-value
Age at time of diagnosis, y	< 47 (*n* = 143)	1 (ref)		1 (ref)	
	≥47 (*n* = 126)	0.563 (0.279–1.132)	0.107	0.470 (0.221–0.998)	0.050*
Education level	Higher (university, graduate school) (*n* = 73)	1 (ref)		1 (ref)	
	Lower (high school, vocational school, junior college) (*n* = 196)	3.881 (1.330–11.325)	0.013	3.802 (1.233–11.729)	0.020*
Cancer stage	Early (0, I) (*n* = 163)	1 (ref)		1 (ref)	
	Advanced (II–IV) (*n* = 106)	2.110 (1.071–4.158)	0.031	1.989 (0.875–4.518)	0.101
Surgery	No (*n* = 19)	1 (ref)		1 (ref)	
	Yes (*n* = 250)	3.327 (0.432–25.649)	0.249	3.115 (0.357–27.154)	0.304
Chemotherapy	No (*n* = 70)	1 (ref)		1 (ref)	
	Yes (*n* = 199)	1.251 (0.563–2.777)	0.583	0.923 (0.345–2.468)	0.873
Radiotherapy	No (*n* = 95)	1 (ref)		1 (ref)	
	Yes (*n* = 174)	0.699 (0.353–1.385)	0.304	0.746 (0.345–1.611)	0.455
Type of employment	Permanent (*n* = 117)	1 (ref)		1 (ref)	
	Non-permanent (*n* = 152)	1.049 (0.532–2.068)	0.891	0.655 (0.306–1.402)	0.276
Occupation type	Office work (*n* = 160)	1 (ref)		1 (ref)	
	Non-office work (*n* = 109)	2.250 (1.138–4.447)	0.020	1.898 (0.906–3.973)	0.089
Sick leave	No (*n* = 174)	1 (ref)		1 (ref)	
	Yes (*n* = 95)	2.950 (1.485–5.859)	0.002	2.514 (1.202–5.261)	0.014*

* < 0.05

Of 229 BCSs who had not resigned (at 1 year after diagnosis), 72 (31.3%) took SL because of cancer treatment (Table 3). Multivariable analysis regarding the risk factors for taking SL demonstrated significance only for surgery (OR: 8.311; 95%CI: 1.007–68.621; *p* = 0.049), as shown in Table 4.

**Table 3:** Basic characteristics of patients who did not resign after breast cancer diagnosis (*n* = 229)

		Sick leave	No sick leave	*p*-value
		n (%)	n (%)	
Age at time of diagnosis, y	< 47	37 (51.4)	80 (51.0)	0.951
	≥47	35 (48.6)	77 (49.0)	
Education level	Higher (university, graduate school)	19 (26.4)	50 (31.8)	0.403
	Lower (high school, vocational school, junior college)	53 (73.6)	107 (68.2)	
Cancer stage	Early (0, I)	39 (54.2)	106 (67.5)	0.052
	Advanced (II–IV)	33 (45.8)	51 (32.5)	
Surgery	No	1 (1.4)	17 (10.8)	0.015*
	Yes	71 (98.6)	140 (89.2)	
Chemotherapy	No	16 (22.2)	45 (28.7)	0.306
	Yes	56 (77.8)	112 (68.6)	
Radiotherapy	No	25 (34.7)	53 (33.8)	0.886
	Yes	47 (65.3)	104 (66.2)	
Employment status	Permanent	26 (36.1)	74 (47.1)	0.118
	Non-permanent	46 (63.9)	83 (52.9)	
Occupation type	Office work	39 (54.2)	104 (66.2)	0.080
	Non-office work	33 (45.8)	53 (33.8)	

* < 0.05

**Table 4:** Univariable and multivariable logistic regression analysis regarding risk factors for taking sick leave

		Univariable		Multivariable	
		OR (95% CI)	*p*-value	OR (95% CI)	*p*-value
Age at time of diagnosis, y	< 47 (*n* = 117)	1 (ref)		1 (ref)	
	≥47 (*n* = 112)	0.983 (0.562–1.717)	0.951	0.777 (0.432–1.396)	0.398
Education level	Higher (university, graduate school) (*n* = 69)	1 (ref)		1 (ref)	
	Lower (high school, vocational school, junior college) (*n* = 160)	1.303 (0.700–2.429)	0.404	1.202 (0.624–2.316)	0.583
Cancer stage	Early (0, I) (*n* = 145)	1 (ref)		1 (ref)	
	Advanced (II–IV) (*n* = 84)	1.759 (0.993–3.114)	0.053	1.545 (0.818–2.919)	0.180
Surgery	No (*n* = 18)	1 (ref)		1 (ref)	
	Yes (*n* = 211)	8.621 (1.125–66.099)	0.038	8.311 (1.007–68.621)	0.049*
Chemotherapy	No (*n* = 61)	1 (ref)		1 (ref)	
	Yes (*n* = 168)	1.406 (0.731–2.706)	0.307	0.969 (0.454–2.069)	0.935
Radiotherapy	No (*n* = 78)	1 (ref)		1 (ref)	
	Yes (*n* = 151)	0.958 (0.533–1.724)	0.886	0.884 (0.467–1.672)	0.704
Employment status	Permanent (*n* = 100)	1 (ref)		1 (ref)	
	Non-permanent (*n* = 129)	1.577 (0.889–2.800)	0.120	1.373 (0.751–2.508)	0.303
Occupation type	Office work (*n* = 143)	1 (ref)		1 (ref)	
	Non-office work (*n* = 86)	1.660 (0.939–2.935)	0.081	1.457 (0.793–2.677)	0.225

* < 0.05

## Discussion

To the best of our knowledge, other than Saito et al. [18], who carried out a cross-sectional study (*n* = 105) that investigated work-related as opposed to clinical factors (e.g., cancer stage, surgery), this is the first study to investigate predictors of job resignation and SL among BCSs in Japan. We found that 14.9% of the BCSs in this study quit their jobs at least 1 year after being diagnosed with breast cancer. In addition, the post-cancer diagnosis resignation rate differed significantly according to education level, cancer stage, and occupational type. A systematic review reported that CSs were more likely to be unemployed than were healthy controls (33.8% vs. 15.2%, respectively; pooled relative risk: 1.37, 16], which suggests that developed countries support CSs to avoid potentially high numbers of resignations [20]. The resignation rate (14.9%) of BCSs in this study was lower than that reported in the previous systematic review [16]. Endo et al. [20] reported that resignation rates were quite low among total cancer in Japan (12.4%), where it is very difficult and uncommon for employers to fire employees. The Labor Contract Act of Japan states the following: “A dismissal shall, if it lacks objectively reasonable grounds and is not considered to be appropriate in general societal terms, be treated as an abuse of right and be invalid” [20].

This study found that age at diagnosis, lower education level, and taking SL were predictors of resignation after breast cancer diagnosis; predictors of taking SL were limited to having undergone surgery. We therefore speculated that being highly educated or taking SL might be confounded by being able to access the SL scheme for workers at larger companies easily, as the SL system is better established in larger than in smaller companies [20]. Since the results from this study might depend on the availability of SL, the relationship between the length of SL or the work environment and resignation after breast cancer diagnosis should be studied in the future.

Regarding predictors of resignation after breast cancer diagnosis, first, our findings indicated that younger BCSs resigned more frequently than their older counterparts, in accordance with previous studies that argue that young BCSs have a higher risk of losing paid employment because breast cancer and its associated treatment are often more aggressive at a younger age, suggesting that young BCSs may experience more severe long-term adverse effects, including those that are work-related (or related to substance of work) [22, 23]. In addition, older people may have more knowledge and technology related to the companies and work compared with younger people [16, 20]. Our data suggest that older BCSs may be more reticent to resign, given the typical age-associated difficulties in finding new employment. However, Fantoni et al. [24] reported that older age was associated with difficulty continuing work and a higher risk of unemployment. Further studies exploring the reasons behind resignation are therefore warranted.

Second, patients with lower compared with higher educational attainment were found to be at higher risk for resignation. This finding is consistent with previous studies of non-Asian populations [12, 25–28]. However, a comparison of resignation rates with studies from other countries warrants careful consideration, given the important differences in socioenvironmental factors, including the widely differing regulation of medical leave provision by national systems and the availability of company-based health care resources [29]. In addition, income has been shown to be correlated with education level: lower income has been found to be associated with an increased likelihood of resignation and unemployment among BCSs [12, 25, 30–32]. Furthermore, educational attainment is likely related to occupation type, with less educated individuals more likely to be working in physically demanding jobs such as manual labor [33]. A MHLW survey in Japan found that people with lower education levels were more likely to have physically demanding jobs such those in the hospitality and wholesale and retail trade industries [34]. Employees with more physically demanding jobs such as manual labor and blue-collar work are more susceptible to resignation [12, 25, 28, 35, 36]. Petersson et al. [37, 38] reported that higher education level was related to greater dedication to work, and that RTW was earlier in patients who valued their work more highly.

Third, our results indicated that the risk of resignation was substantially higher among BCSs who took SL after breast cancer diagnosis than among those who did not. These findings are consistent with previous studies that showed a correlation between length of SL and RTW, with longer SL making RTW and continued employment more difficult [39, 40]. Conversely, Azarkish et al. [27] found no relationship between taking SL and job loss. Longer SL is reported to be associated with more invasive treatment, advanced breast cancer, and economic deprivation, all of which are factors related to unemployment [25, 40, 41].

Regarding predictors of taking SL, our findings indicated that BCSs who had undergone surgery took SL more frequently than those who had undergone nonsurgical interventions. The distinction between BCSs who undergo surgery and those who do not suggests a relation to cancer stage (early or advanced) because almost all BCSs undergo surgery, except for those with stage IV cancer, in which distant metastasis is apparent. Previous studies have reported that breast cancer surgery is associated with SL lasting 1 month or longer [42, 43], and that the median duration of hospitalization among BCSs in Japan is about 6.79–10.37 days [44]. Surgical treatment may result in challenging sequelae, including scar pain, fatigue, lymphedema, and reduced range of motion, particularly in the arm and chest region; these symptoms increase the time to RTW and are related to unemployment [45]. Wennman-Larsen et al. [46] reported that arm morbidity shortly after surgery affected 10% of BCSs, and that 60% of these patients were on SL; SL was linked to arm morbidity, axillary clearance, and strenuous work posture. More invasive surgery is also related to more advanced breast cancer, which leads to more severe sequelae and longer SL [41]. Petersson et al. [47] proposed that various side effects related to surgery impair work capacity and lead to longer SL in occupations requiring strenuous work postures.

This study did have some limitations. First, recall bias is possible given the nature of the self-report questionnaire design. In particular, as cognitive function may be adversely affected by some forms of treatment, some of the respondents may have been unable to remember when they had been diagnosed with breast cancer or to report how their work had changed after diagnosis. Second, this study was affected by survivorship bias, a form of selection bias, as BCSs who died before completing the questionnaire were excluded. Because BCSs who had been diagnosed with breast cancer within 1 year prior to participation in this study were excluded, we speculate that the resignation rate among BCSs was underestimated because of the death of patients who had left their jobs soon after diagnosis, especially in cases of advanced-stage disease. In addition, younger patients may have felt more comfortable than older patients given the online delivery and design of the survey. Third, SL systems depend on their company rules, so it might be difficult to discuss the risk factors of resignation more strictly. However, as the number of days of annual paid leave is stipulated by the Labor Standards Act [19], and the SL process after using up annual paid leave is common among all Japanese companies, it seems that there is less effect on the risk of SL among BCSs among different companies. Fourth, the response rate was relatively low (10.4%) because a response was required within 2 days of receiving the questionnaire. It might be possible to increase the response rate by extending the response period. Finally, the sample size was small because a large number of respondents were ultimately excluded from analysis; further large-scale investigations are required to corroborate our results.

As a future task, while we provided little clinical implications based on the findings of this research, a prospective cohort study (such as an RTW intervention study) involving working BCSs in Japan is needed to clarify the association between clinical factors (symptoms) and work-related factors among BCSs.

## Conclusion

This study investigated the risk factors for resignation and taking SL after breast cancer diagnosis in Japan. The results indicated that 14.9% of the women diagnosed with BC from the January 17–18, 2018 registry and who were employed at the time of diagnosis quit their job at least 1 year after diagnosis. Younger age at breast cancer diagnosis, a lower education level, and taking SL were identified as predictors of resignation after breast cancer diagnosis, while surgery was associated with the highest risk of taking SL.

## Supplementary Information

**Additional file 1.** A working condition questionnaire for breast cancer survivors

## Data Availability

The datasets analyzed during the current study are not publicly available. In addition, due to security aspects, data can be analyzed only in a safe place. Researchers may contact the corresponding Author for questions concerning the data.

## Abbreviations

BCSs: Breast cancer survivors
CSs: Cancer survivors
QOL: Quality of life
RTW: Return to work
SL: Sick leave
