# Supplementary material for: Predictors of resignation and sick leave after cancer diagnosis among Japanese breast cancer survivors: a cross-sectional study
Source: BMC Public Health. 2021 Jan 14;21:138. doi: 10.1186/s12889-021-10168-2 (PMC7809813; doi:10.1186/s12889-021-10168-2)
Supplement: Supplementary file 1 — Additional file 1. A working condition questionnaire for breast cancer survivors [file 12889_2021_10168_MOESM1_ESM.docx]

Additional file 1. A working condition questionnaire for breast cancer survivors

(A web-based retrospective cross-sectional survey was conducted on breast cancer survivors using data from a 2018 Japanese national research project (Endo-Han) commissioned by the Ministry of Health, Labour and Welfare (MHLW) of Japan. The project developed the following questionnaire)

Q: What is your birthday?

Date/Month/Year

Q: What is the highest level of education you have achieved?

• Middle school

• High school

• Junior college

• Vocational college

• University

• Graduate school

Q: What is your marriage status?

• Unmarried

• Married

• Divorced

• Widowed

Q: When were you diagnosed with breast cancer?

• Month/Year

Q: What is the stage of your breast cancer?

• Stage 0

• Stage I

• Stage II

• Stage III

• Stage IV

Q: Have you had a breast cancer surgery?

• No

• Yes

Q: Have you had a chemotherapy?

• No

• Yes

Q: Have you had a radiation therapy to treat the breast cancer?

• No

• Yes

Q: Please circle all [diseases you had (other than breast cancer)] in the past.

• Cancer (type)

• Stroke

• Other

• No other disease

Q: Are you currently continuing with the breast cancer treatment?

• Yes

• No

Q: Were you working when you were diagnosed with the breast cancer?

• Yes

• No

Q: (This question is for those who were working when diagnosed with the breast cancer.)

What happened to work after the diagnosis? Please choose the answer that fits the best.

• I continued to work without taking any time off.

• I used the paid leave for treatment, then returned to work.

• Since the paid leave was not enough, I took a sick leave, leave of absence, and so on for treatment, then returned to work (duration: months).

• I have been working since the diagnosis without any time off (duration: months).

• I quit work after the diagnosis (without going back to work at all) (duration: months).

Q: This question is for those who said, “I returned to work after the breast cancer treatment”.

What happened after you returned to (the previous workplace)?

• I still work at the same workplace after the breast cancer treatment ( months since I returned to work).

• I quit work after returning to work following the breast cancer treatment (quit after months following the return to work).

• I needed to recuperate after returning to work from the breast cancer treatment (I needed to recuperate months after returning to work).

Q: What is your occupational position? *At the workplace you were at when diagnosed with the breast cancer.

• Management

• Non-management

Q: What was your employment classification? *At the workplace you were at when diagnosed with the breast cancer.

• Full time

• Part time

Q: What was your occupation type? *At the workplace you were at when diagnosed with the breast cancer.

• Office work

• Non-office work

Thank you for taking your time.

(Contact: Executive office of Endo-Han)

Research supervisor: Motoki Endo

[mo-endo@juntendo.ac.jp](mailto:mo-endo@juntendo.ac.jp)

Department of Public Health, Juntendo University

2 Chome 1-1, Hongo, Bunkyo Ward, Tokyo Prefecture, 113-8421

Phone: 03-5802-1049

© Macromill,Inc. All Rights Reserved.
